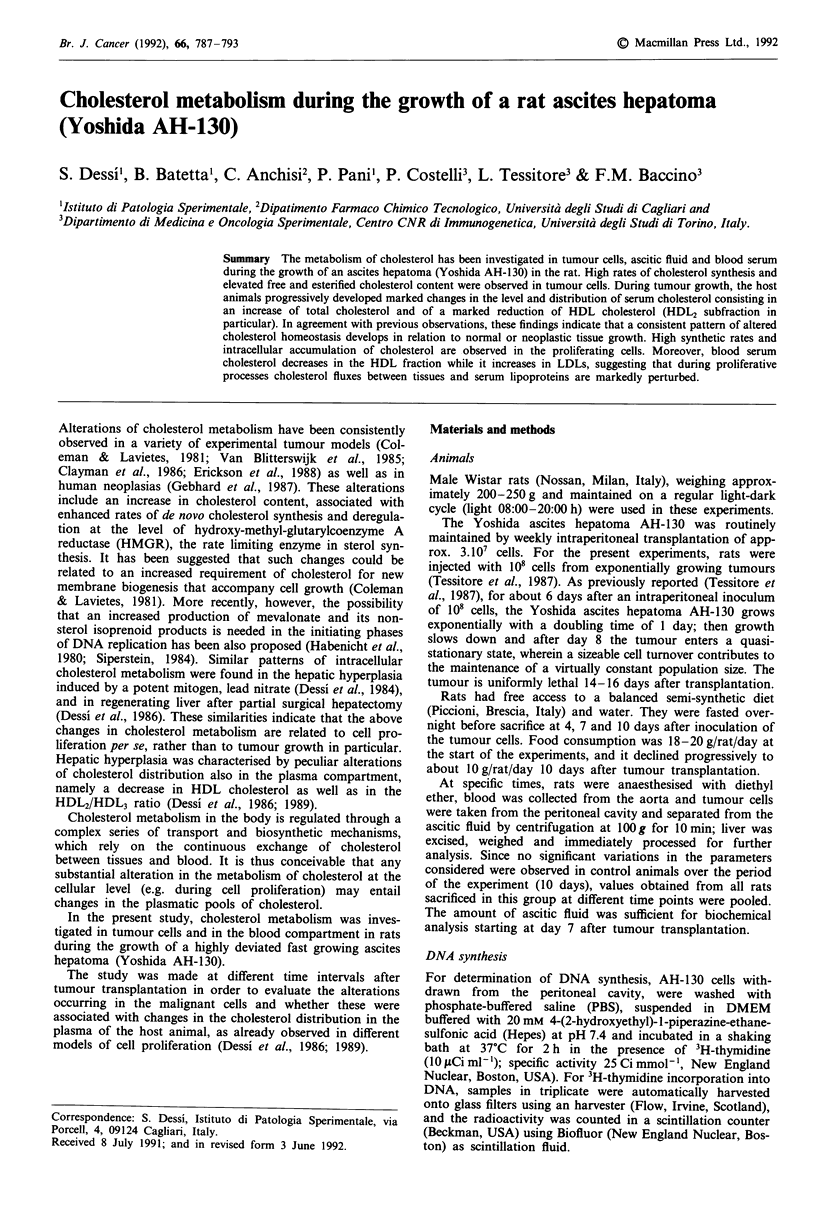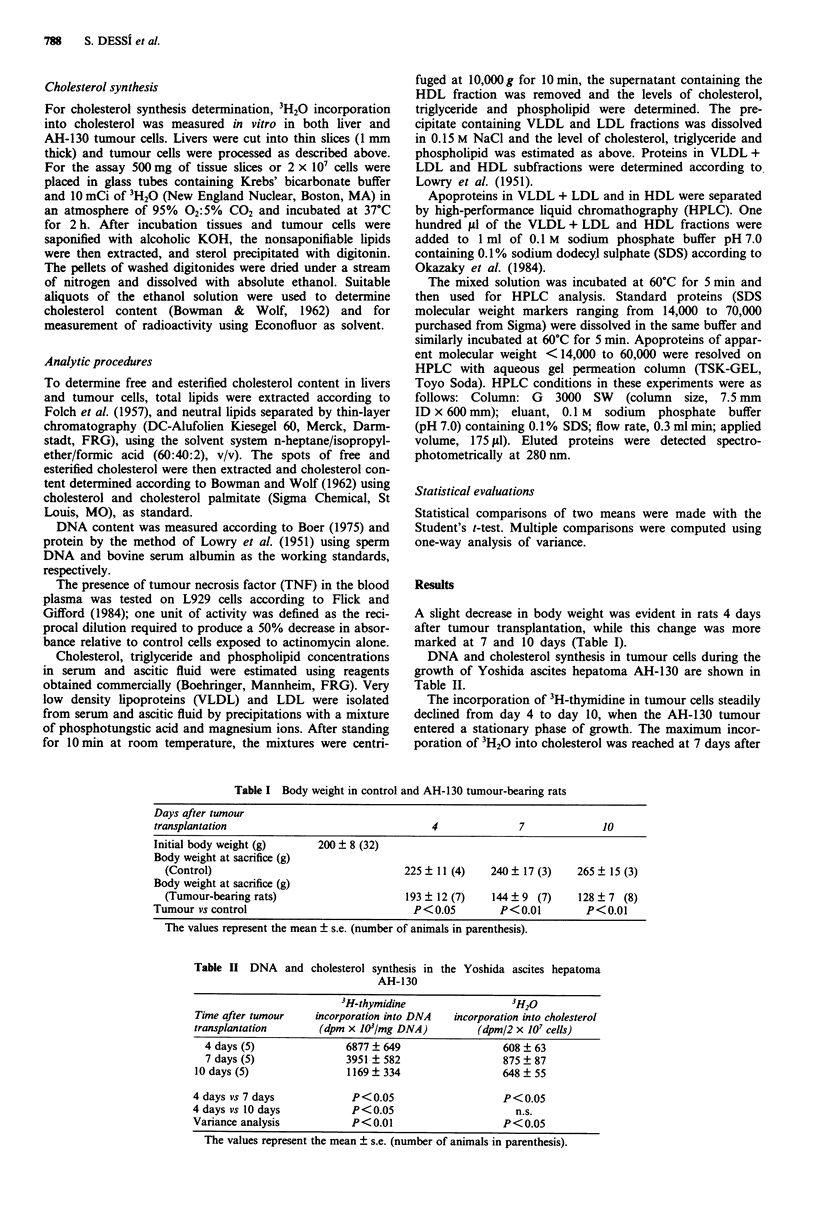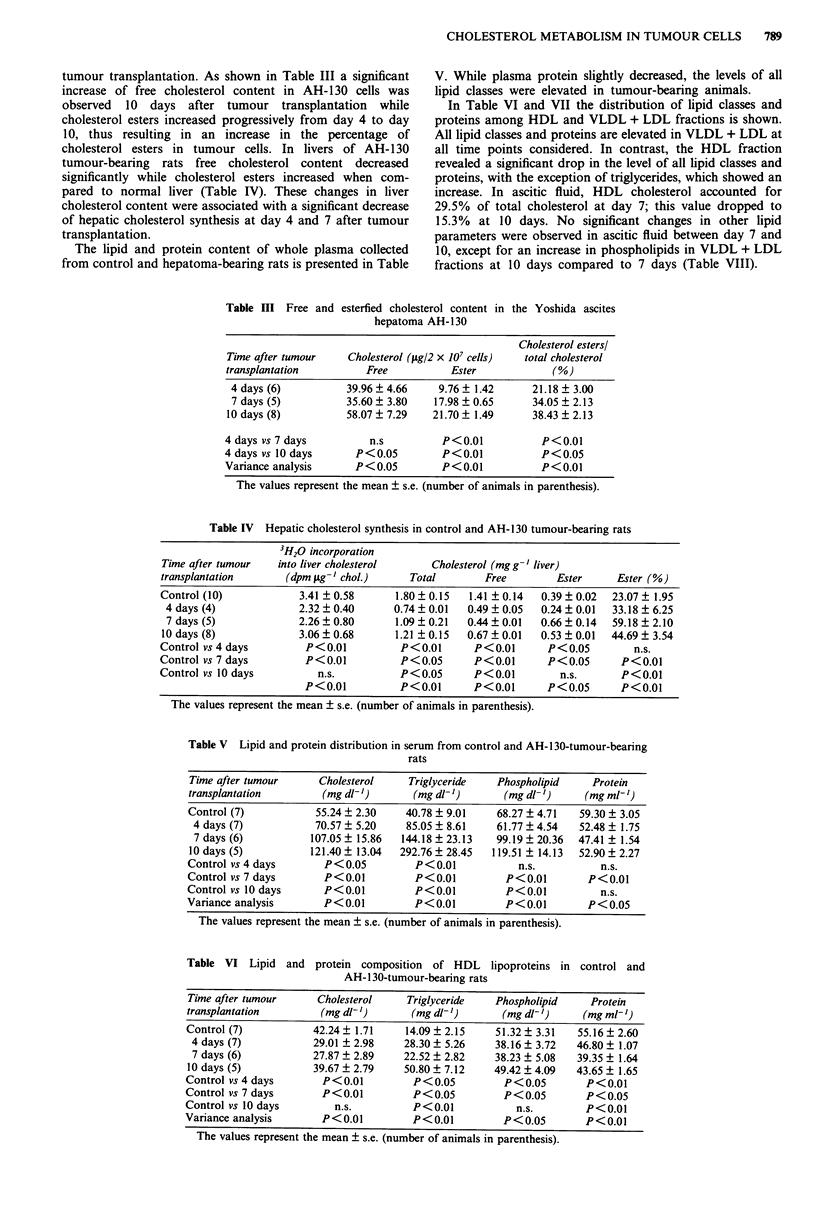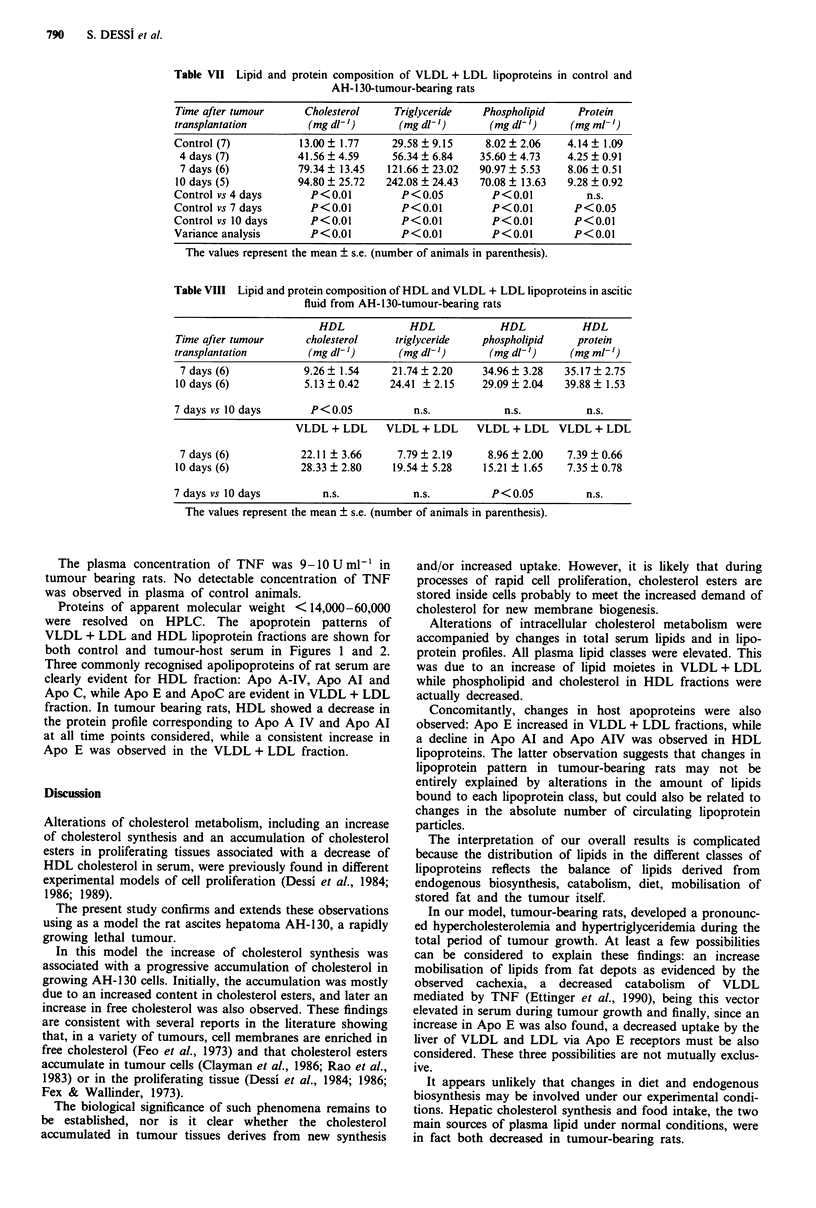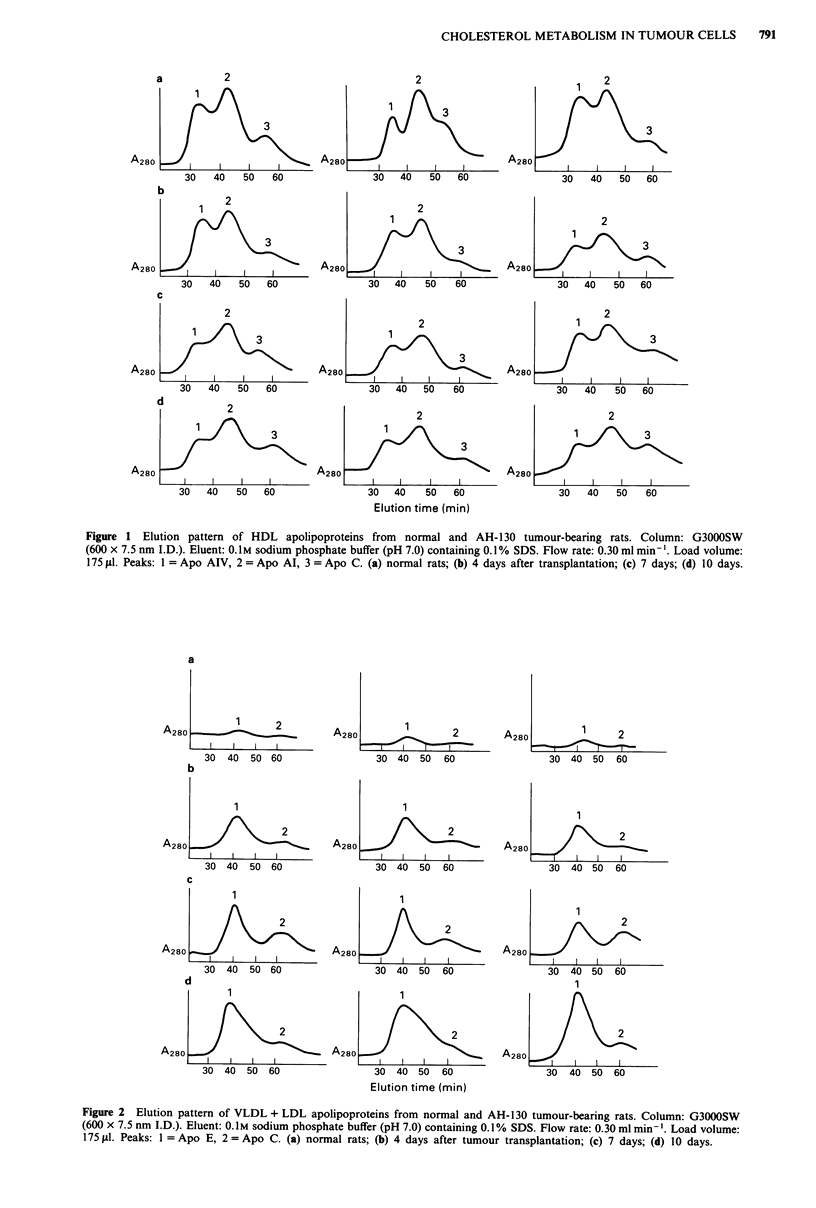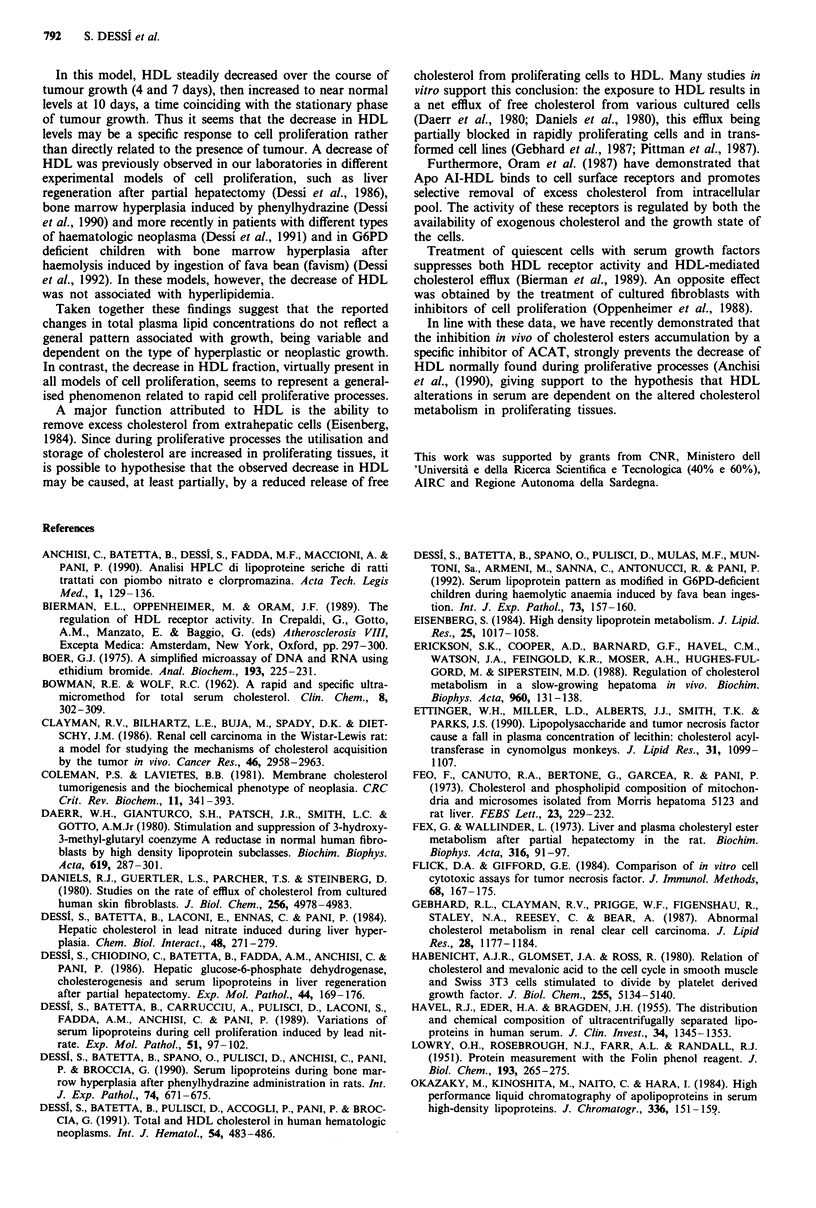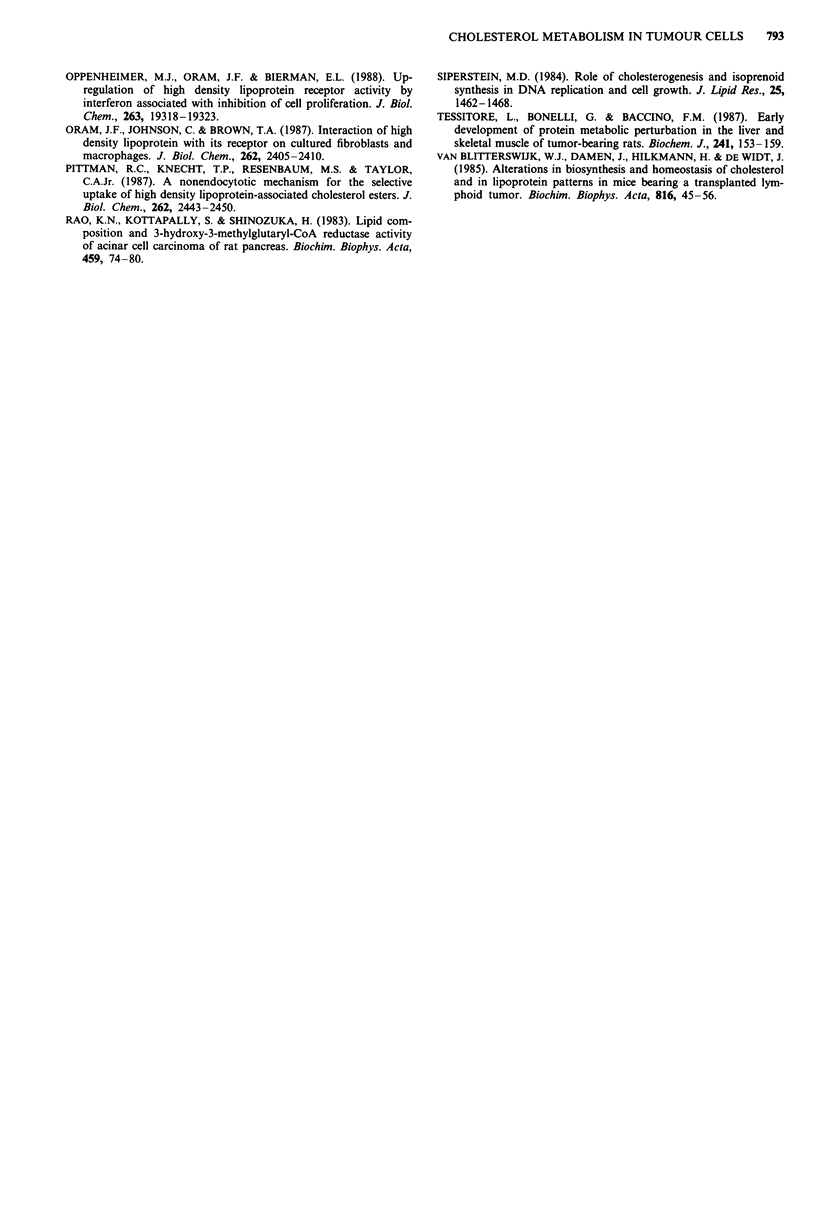# Cholesterol metabolism during the growth of a rat ascites hepatoma (Yoshida AH-130).

**DOI:** 10.1038/bjc.1992.361

**Published:** 1992-11

**Authors:** S. Dessí, B. Batetta, C. Anchisi, P. Pani, P. Costelli, L. Tessitore, F. M. Baccino

**Affiliations:** Istituto di Patologia Sperimentale, Università degli Studi di Cagliari, Italy.

## Abstract

The metabolism of cholesterol has been investigated in tumour cells, ascitic fluid and blood serum during the growth of an ascites hepatoma (Yoshida AH-130) in the rat. High rates of cholesterol synthesis and elevated free and esterified cholesterol content were observed in tumour cells. During tumour growth, the host animals progressively developed marked changes in the level and distribution of serum cholesterol consisting in an increase of total cholesterol and of a marked reduction of HDL cholesterol (HDL2 subfraction in particular). In agreement with previous observations, these findings indicate that a consistent pattern of altered cholesterol homeostasis develops in relation to normal or neoplastic tissue growth. High synthetic rates and intracellular accumulation of cholesterol are observed in the proliferating cells. Moreover, blood serum cholesterol decreases in the HDL fraction while it increases in LDLs, suggesting that during proliferative processes cholesterol fluxes between tissues and serum lipoproteins are markedly perturbed.


Br. J. Cancer (1992), 66, 787-793 © Macmillan Press Ltd., 1992

### Cholesterol metabolism during the growth of a rat ascites hepatoma (Yoshida AH-130)

S. Dessí[1], B. Batetta[1], C. Anchisi[2], P. Pani[1], P. Costelli[3], L. Tessitore[3] & F.M. Baccino[3]

[1]*Istituto di Patologia Sperimentale,* [2]*Dipatimento Farmaco Chimico Tecnologico, Università degli Studi di Cagliari and* [3]*Dipartimento di Medicina e Oncologia Sperimentale, Centro CNR di Immunogenetica, Università degli Studi di Torino, Italy.*

**Summary** The metabolism of cholesterol has been investigated in tumour cells, ascitic fluid and blood serum during the growth of an ascites hepatoma (Yoshida AH-130) in the rat. High rates of cholesterol synthesis and elevated free and esterified cholesterol content were observed in tumour cells. During tumour growth, the host animals progressively developed marked changes in the level and distribution of serum cholesterol consisting in an increase of total cholesterol and of a marked reduction of HDL cholesterol ($HDL_2$ subfraction in particular). In agreement with previous observations, these findings indicate that a consistent pattern of altered cholesterol homeostasis develops in relation to normal or neoplastic tissue growth. High synthetic rates and intracellular accumulation of cholesterol are observed in the proliferating cells. Moreover, blood serum cholesterol decreases in the HDL fraction while it increases in LDLs, suggesting that during proliferative processes cholesterol fluxes between tissues and serum lipoproteins are markedly perturbed.

Alterations of cholesterol metabolism have been consistently observed in a variety of experimental tumour models (Coleman & Lavietes, 1981; Van Blitterswijk *et al.*, 1985; Clayman *et al.*, 1986; Erickson *et al.*, 1988) as well as in human neoplasias (Gebhard *et al.*, 1987). These alterations include an increase in cholesterol content, associated with enhanced rates of *de novo* cholesterol synthesis and deregulation at the level of hydroxy-methyl-glutarylcoenzyme A reductase (HMGR), the rate limiting enzyme in sterol synthesis. It has been suggested that such changes could be related to an increased requirement of cholesterol for new membrane biogenesis that accompany cell growth (Coleman & Lavietes, 1981). More recently, however, the possibility that an increased production of mevalonate and its non-sterol isoprenoid products is needed in the initiating phases of DNA replication has been also proposed (Habenicht *et al.*, 1980; Siperstein, 1984). Similar patterns of intracellular cholesterol metabolism were found in the hepatic hyperplasia induced by a potent mitogen, lead nitrate (Dessí *et al.*, 1984), and in regenerating liver after partial surgical hepatectomy (Dessí *et al.*, 1986). These similarities indicate that the above changes in cholesterol metabolism are related to cell proliferation *per se*, rather than to tumour growth in particular. Hepatic hyperplasia was characterised by peculiar alterations of cholesterol distribution also in the plasma compartment, namely a decrease in HDL cholesterol as well as in the $HDL_2/HDL_3$ ratio (Dessí *et al.*, 1986; 1989).

Cholesterol metabolism in the body is regulated through a complex series of transport and biosynthetic mechanisms, which rely on the continuous exchange of cholesterol between tissues and blood. It is thus conceivable that any substantial alteration in the metabolism of cholesterol at the cellular level (e.g. during cell proliferation) may entail changes in the plasmatic pools of cholesterol.

In the present study, cholesterol metabolism was investigated in tumour cells and in the blood compartment in rats during the growth of a highly deviated fast growing ascites hepatoma (Yoshida AH-130).

The study was made at different time intervals after tumour transplantation in order to evaluate the alterations occurring in the malignant cells and whether these were associated with changes in the cholesterol distribution in the plasma of the host animal, as already observed in different models of cell proliferation (Dessí *et al.*, 1986; 1989).

Correspondence: S. Dessí, Istituto di Patologia Sperimentale, via Porcell, 4, 09124 Cagliari, Italy.
Received 8 July 1991; and in revised form 3 June 1992.

### Materials and methods

### *Animals*

Male Wistar rats (Nossan, Milan, Italy), weighing approximately 200-250 g and maintained on a regular light-dark cycle (light 08:00-20:00 h) were used in these experiments.

The Yoshida ascites hepatoma AH-130 was routinely maintained by weekly intraperitoneal transplantation of approx. $3.10^7$ cells. For the present experiments, rats were injected with $10^8$ cells from exponentially growing tumours (Tessitore *et al.*, 1987). As previously reported (Tessitore *et al.*, 1987), for about 6 days after an intraperitoneal inoculum of $10^8$ cells, the Yoshida ascites hepatoma AH-130 grows exponentially with a doubling time of 1 day; then growth slows down and after day 8 the tumour enters a quasi-stationary state, wherein a sizeable cell turnover contributes to the maintenance of a virtually constant population size. The tumour is uniformly lethal 14-16 days after transplantation.

Rats had free access to a balanced semi-synthetic diet (Piccioni, Brescia, Italy) and water. They were fasted overnight before sacrifice at 4, 7 and 10 days after inoculation of the tumour cells. Food consumption was 18-20 g/rat/day at the start of the experiments, and it declined progressively to about 10 g/rat/day 10 days after tumour transplantation.

At specific times, rats were anaesthesised with diethyl ether, blood was collected from the aorta and tumour cells were taken from the peritoneal cavity and separated from the ascitic fluid by centrifugation at 100 ***g*** for 10 min; liver was excised, weighed and immediately processed for further analysis. Since no significant variations in the parameters considered were observed in control animals over the period of the experiment (10 days), values obtained from all rats sacrificed in this group at different time points were pooled. The amount of ascitic fluid was sufficient for biochemical analysis starting at day 7 after tumour transplantation.

### *DNA synthesis*

For determination of DNA synthesis, AH-130 cells withdrawn from the peritoneal cavity, were washed with phosphate-buffered saline (PBS), suspended in DMEM buffered with 20 mM 4-(2-hydroxyethyl)-1-piperazine-ethane-sulfonic acid (Hepes) at pH 7.4 and incubated in a shaking bath at 37°C for 2 h in the presence of $^3$H-thymidine (10 $\mu$Ci ml$^{-1}$); specific activity 25 Ci mmol$^{-1}$, New England Nuclear, Boston, USA). For $^3$H-thymidine incorporation into DNA, samples in triplicate were automatically harvested onto glass filters using an harvester (Flow, Irvine, Scotland), and the radioactivity was counted in a scintillation counter (Beckman, USA) using Biofluor (New England Nuclear, Boston) as scintillation fluid.

*Cholesterol synthesis*

For cholesterol synthesis determination, $^3H_2O$ incorporation into cholesterol was measured *in vitro* in both liver and AH-130 tumour cells. Livers were cut into thin slices (1 mm thick) and tumour cells were processed as described above. For the assay 500 mg of tissue slices or $2 \times 10^7$ cells were placed in glass tubes containing Krebs' bicarbonate buffer and 10 mCi of $^3H_2O$ (New England Nuclear, Boston, MA) in an atmosphere of 95% $O_2$:5% $CO_2$ and incubated at 37°C for 2 h. After incubation tissues and tumour cells were saponified with alcoholic KOH, the nonsaponifiable lipids were then extracted, and sterol precipitated with digitonin. The pellets of washed digitonides were dried under a stream of nitrogen and dissolved with absolute ethanol. Suitable aliquots of the ethanol solution were used to determine cholesterol content (Bowman & Wolf, 1962) and for measurement of radioactivity using Econofluor as solvent.

*Analytic procedures*

To determine free and esterified cholesterol content in livers and tumour cells, total lipids were extracted according to Folch *et al.* (1957), and neutral lipids separated by thin-layer chromatography (DC-Alufolien Kiesegel 60, Merck, Darmstadt, FRG), using the solvent system n-heptane/isopropyl-ether/formic acid (60:40:2), v/v). The spots of free and esterified cholesterol were then extracted and cholesterol content determined according to Bowman and Wolf (1962) using cholesterol and cholesterol palmitate (Sigma Chemical, St Louis, MO), as standard.

DNA content was measured according to Boer (1975) and protein by the method of Lowry *et al.* (1951) using sperm DNA and bovine serum albumin as the working standards, respectively.

The presence of tumour necrosis factor (TNF) in the blood plasma was tested on L929 cells according to Flick and Gifford (1984); one unit of activity was defined as the reciprocal dilution required to produce a 50% decrease in absorbance relative to control cells exposed to actinomycin alone.

Cholesterol, triglyceride and phospholipid concentrations in serum and ascitic fluid were estimated using reagents obtained commercially (Boehringer, Mannheim, FRG). Very low density lipoproteins (VLDL) and LDL were isolated from serum and ascitic fluid by precipitations with a mixture of phosphotungstic acid and magnesium ions. After standing for 10 min at room temperature, the mixtures were centrifuged at 10,000 *g* for 10 min, the supernatant containing the HDL fraction was removed and the levels of cholesterol, triglyceride and phospholipid were determined. The precipitate containing VLDL and LDL fractions was dissolved in 0.15 M NaCl and the level of cholesterol, triglyceride and phospholipid was estimated as above. Proteins in VLDL + LDL and HDL subfractions were determined according to Lowry *et al.* (1951).

Apoproteins in VLDL + LDL and in HDL were separated by high-performance liquid chromathography (HPLC). One hundred μl of the VLDL + LDL and HDL fractions were added to 1 ml of 0.1 M sodium phosphate buffer pH 7.0 containing 0.1% sodium dodecyl sulphate (SDS) according to Okazaky *et al.* (1984).

The mixed solution was incubated at 60°C for 5 min and then used for HPLC analysis. Standard proteins (SDS molecular weight markers ranging from 14,000 to 70,000 purchased from Sigma) were dissolved in the same buffer and similarly incubated at 60°C for 5 min. Apoproteins of apparent molecular weight <14,000 to 60,000 were resolved on HPLC with aqueous gel permeation column (TSK-GEL, Toyo Soda). HPLC conditions in these experiments were as follows: Column: G 3000 SW (column size, 7.5 mm ID × 600 mm); eluant, 0.1 M sodium phosphate buffer (pH 7.0) containing 0.1% SDS; flow rate, 0.3 ml min; applied volume, 175 μl). Eluted proteins were detected spectrophotometrically at 280 nm.

*Statistical evaluations*

Statistical comparisons of two means were made with the Student's *t*-test. Multiple comparisons were computed using one-way analysis of variance.

### Results

A slight decrease in body weight was evident in rats 4 days after tumour transplantation, while this change was more marked at 7 and 10 days (Table I).

DNA and cholesterol synthesis in tumour cells during the growth of Yoshida ascites hepatoma AH-130 are shown in Table II.

The incorporation of $^3$H-thymidine in tumour cells steadily declined from day 4 to day 10, when the AH-130 tumour entered a stationary phase of growth. The maximum incorporation of $^3H_2O$ into cholesterol was reached at 7 days after

**Table I** Body weight in control and AH-130 tumour-bearing rats

| *Days after tumour transplantation* | | *4* | *7* | *10* |
|---|---|---|---|---|
| Initial body weight (g) | 200 ± 8 (32) | | | |
| Body weight at sacrifice (g) (Control) | | 225 ± 11 (4) | 240 ± 17 (3) | 265 ± 15 (3) |
| Body weight at sacrifice (g) (Tumour-bearing rats) | | 193 ± 12 (7) | 144 ± 9 (7) | 128 ± 7 (8) |
| Tumour *vs* control | | $P<0.05$ | $P<0.01$ | $P<0.01$ |

The values represent the mean ± s.e. (number of animals in parenthesis).

**Table II** DNA and cholesterol synthesis in the Yoshida ascites hepatoma AH-130

| *Time after tumour transplantation* | *$^3$H-thymidine incorporation into DNA (dpm × $10^3$/mg DNA)* | *$^3H_2O$ incorporation into cholesterol (dpm/2 × $10^7$ cells)* |
|---|---|---|
| 4 days (5) | 6877 ± 649 | 608 ± 63 |
| 7 days (5) | 3951 ± 582 | 875 ± 87 |
| 10 days (5) | 1169 ± 334 | 648 ± 55 |
| 4 days *vs* 7 days | $P<0.05$ | $P<0.05$ |
| 4 days *vs* 10 days | $P<0.05$ | n.s. |
| Variance analysis | $P<0.01$ | $P<0.05$ |

The values represent the mean ± s.e. (number of animals in parenthesis).

tumour transplantation. As shown in Table III a significant increase of free cholesterol content in AH-130 cells was observed 10 days after tumour transplantation while cholesterol esters increased progressively from day 4 to day 10, thus resulting in an increase in the percentage of cholesterol esters in tumour cells. In livers of AH-130 tumour-bearing rats free cholesterol content decreased significantly while cholesterol esters increased when compared to normal liver (Table IV). These changes in liver cholesterol content were associated with a significant decrease of hepatic cholesterol synthesis at day 4 and 7 after tumour transplantation.

The lipid and protein content of whole plasma collected from control and hepatoma-bearing rats is presented in Table V. While plasma protein slightly decreased, the levels of all lipid classes were elevated in tumour-bearing animals.

In Table VI and VII the distribution of lipid classes and proteins among HDL and VLDL + LDL fractions is shown. All lipid classes and proteins are elevated in VLDL + LDL at all time points considered. In contrast, the HDL fraction revealed a significant drop in the level of all lipid classes and proteins, with the exception of triglycerides, which showed an increase. In ascitic fluid, HDL cholesterol accounted for 29.5% of total cholesterol at day 7; this value dropped to 15.3% at 10 days. No significant changes in other lipid parameters were observed in ascitic fluid between day 7 and 10, except for an increase in phospholipids in VLDL + LDL fractions at 10 days compared to 7 days (Table VIII).

**Table III** Free and esterfied cholesterol content in the Yoshida ascites hepatoma AH-130

| *Time after tumour transplantation* | *Cholesterol (μg/2 × 10$^7$ cells)* Free | Ester | *Cholesterol esters/ total cholesterol (%)* |
|---|---|---|---|
| 4 days (6) | 39.96 ± 4.66 | 9.76 ± 1.42 | 21.18 ± 3.00 |
| 7 days (5) | 35.60 ± 3.80 | 17.98 ± 0.65 | 34.05 ± 2.13 |
| 10 days (8) | 58.07 ± 7.29 | 21.70 ± 1.49 | 38.43 ± 2.13 |
| 4 days *vs* 7 days | n.s | P<0.01 | P<0.01 |
| 4 days *vs* 10 days | P<0.05 | P<0.01 | P<0.05 |
| Variance analysis | P<0.05 | P<0.01 | P<0.01 |

The values represent the mean ± s.e. (number of animals in parenthesis).

**Table IV** Hepatic cholesterol synthesis in control and AH-130 tumour-bearing rats

| *Time after tumour transplantation* | *$^3H_2O$ incorporation into liver cholesterol (dpm μg$^{-1}$ chol.)* | *Cholesterol (mg g$^{-1}$ liver)* Total | Free | Ester | *Ester (%)* |
|---|---|---|---|---|---|
| Control (10) | 3.41 ± 0.58 | 1.80 ± 0.15 | 1.41 ± 0.14 | 0.39 ± 0.02 | 23.07 ± 1.95 |
| 4 days (4) | 2.32 ± 0.40 | 0.74 ± 0.01 | 0.49 ± 0.05 | 0.24 ± 0.01 | 33.18 ± 6.25 |
| 7 days (5) | 2.26 ± 0.80 | 1.09 ± 0.21 | 0.44 ± 0.01 | 0.66 ± 0.14 | 59.18 ± 2.10 |
| 10 days (8) | 3.06 ± 0.68 | 1.21 ± 0.15 | 0.67 ± 0.01 | 0.53 ± 0.01 | 44.69 ± 3.54 |
| Control *vs* 4 days | P<0.01 | P<0.01 | P<0.01 | P<0.05 | n.s. |
| Control *vs* 7 days | P<0.01 | P<0.05 | P<0.01 | P<0.05 | P<0.01 |
| Control *vs* 10 days | n.s. | P<0.05 | P<0.01 | n.s. | P<0.01 |
| | P<0.01 | P<0.01 | P<0.01 | P<0.05 | P<0.01 |

The values represent the mean ± s.e. (number of animals in parenthesis).

**Table V** Lipid and protein distribution in serum from control and AH-130-tumour-bearing rats

| *Time after tumour transplantation* | *Cholesterol (mg dl$^{-1}$)* | *Triglyceride (mg dl$^{-1}$)* | *Phospholipid (mg dl$^{-1}$)* | *Protein (mg ml$^{-1}$)* |
|---|---|---|---|---|
| Control (7) | 55.24 ± 2.30 | 40.78 ± 9.01 | 68.27 ± 4.71 | 59.30 ± 3.05 |
| 4 days (7) | 70.57 ± 5.20 | 85.05 ± 8.61 | 61.77 ± 4.54 | 52.48 ± 1.75 |
| 7 days (6) | 107.05 ± 15.86 | 144.18 ± 23.13 | 99.19 ± 20.36 | 47.41 ± 1.54 |
| 10 days (5) | 121.40 ± 13.04 | 292.76 ± 28.45 | 119.51 ± 14.13 | 52.90 ± 2.27 |
| Control *vs* 4 days | P<0.05 | P<0.01 | n.s. | n.s. |
| Control *vs* 7 days | P<0.01 | P<0.01 | P<0.01 | P<0.01 |
| Control *vs* 10 days | P<0.01 | P<0.01 | P<0.01 | n.s. |
| Variance analysis | P<0.01 | P<0.01 | P<0.01 | P<0.05 |

The values represent the mean ± s.e. (number of animals in parenthesis).

**Table VI** Lipid and protein composition of HDL lipoproteins in control and AH-130-tumour-bearing rats

| *Time after tumour transplantation* | *Cholesterol (mg dl$^{-1}$)* | *Triglyceride (mg dl$^{-1}$)* | *Phospholipid (mg dl$^{-1}$)* | *Protein (mg ml$^{-1}$)* |
|---|---|---|---|---|
| Control (7) | 42.24 ± 1.71 | 14.09 ± 2.15 | 51.32 ± 3.31 | 55.16 ± 2.60 |
| 4 days (7) | 29.01 ± 2.98 | 28.30 ± 5.26 | 38.16 ± 3.72 | 46.80 ± 1.07 |
| 7 days (6) | 27.87 ± 2.89 | 22.52 ± 2.82 | 38.23 ± 5.08 | 39.35 ± 1.64 |
| 10 days (5) | 39.67 ± 2.79 | 50.80 ± 7.12 | 49.42 ± 4.09 | 43.65 ± 1.65 |
| Control *vs* 4 days | P<0.01 | P<0.05 | P<0.05 | P<0.01 |
| Control *vs* 7 days | P<0.01 | P<0.05 | P<0.05 | P<0.05 |
| Control *vs* 10 days | n.s. | P<0.01 | n.s. | P<0.01 |
| Variance analysis | P<0.01 | P<0.01 | P<0.05 | P<0.01 |

The values represent the mean ± s.e. (number of animals in parenthesis).

**Table VII** Lipid and protein composition of VLDL + LDL lipoproteins in control and AH-130-tumour-bearing rats

| *Time after tumour transplantation* | *Cholesterol ($mg\ dl^{-1}$)* | *Triglyceride ($mg\ dl^{-1}$)* | *Phospholipid ($mg\ dl^{-1}$)* | *Protein ($mg\ ml^{-1}$)* |
|---|---|---|---|---|
| Control (7) | 13.00 ± 1.77 | 29.58 ± 9.15 | 8.02 ± 2.06 | 4.14 ± 1.09 |
| 4 days (7) | 41.56 ± 4.59 | 56.34 ± 6.84 | 35.60 ± 4.73 | 4.25 ± 0.91 |
| 7 days (6) | 79.34 ± 13.45 | 121.66 ± 23.02 | 90.97 ± 5.53 | 8.06 ± 0.51 |
| 10 days (5) | 94.80 ± 25.72 | 242.08 ± 24.43 | 70.08 ± 13.63 | 9.28 ± 0.92 |
| Control *vs* 4 days | $P<0.01$ | $P<0.05$ | $P<0.01$ | n.s. |
| Control *vs* 7 days | $P<0.01$ | $P<0.01$ | $P<0.01$ | $P<0.05$ |
| Control *vs* 10 days | $P<0.01$ | $P<0.01$ | $P<0.01$ | $P<0.01$ |
| Variance analysis | $P<0.01$ | $P<0.01$ | $P<0.01$ | $P<0.01$ |

The values represent the mean ± s.e. (number of animals in parenthesis).

**Table VIII** Lipid and protein composition of HDL and VLDL + LDL lipoproteins in ascitic fluid from AH-130-tumour-bearing rats

| *Time after tumour transplantation* | *HDL cholesterol ($mg\ dl^{-1}$)* | *HDL triglyceride ($mg\ dl^{-1}$)* | *HDL phospholipid ($mg\ dl^{-1}$)* | *HDL protein ($mg\ ml^{-1}$)* |
|---|---|---|---|---|
| 7 days (6) | 9.26 ± 1.54 | 21.74 ± 2.20 | 34.96 ± 3.28 | 35.17 ± 2.75 |
| 10 days (6) | 5.13 ± 0.42 | 24.41 ± 2.15 | 29.09 ± 2.04 | 39.88 ± 1.53 |
| 7 days *vs* 10 days | $P<0.05$ | n.s. | n.s. | n.s. |
| | VLDL + LDL | VLDL + LDL | VLDL + LDL | VLDL + LDL |
| 7 days (6) | 22.11 ± 3.66 | 7.79 ± 2.19 | 8.96 ± 2.00 | 7.39 ± 0.66 |
| 10 days (6) | 28.33 ± 2.80 | 19.54 ± 5.28 | 15.21 ± 1.65 | 7.35 ± 0.78 |
| 7 days *vs* 10 days | n.s. | n.s. | $P<0.05$ | n.s. |

The values represent the mean ± s.e. (number of animals in parenthesis).

The plasma concentration of TNF was 9–10 U $ml^{-1}$ in tumour bearing rats. No detectable concentration of TNF was observed in plasma of control animals.

Proteins of apparent molecular weight <14,000–60,000 were resolved on HPLC. The apoprotein patterns of VLDL + LDL and HDL lipoprotein fractions are shown for both control and tumour-host serum in Figures 1 and 2. Three commonly recognised apolipoproteins of rat serum are clearly evident for HDL fraction: Apo A-IV, Apo AI and Apo C, while Apo E and ApoC are evident in VLDL + LDL fraction. In tumour bearing rats, HDL showed a decrease in the protein profile corresponding to Apo A IV and Apo AI at all time points considered, while a consistent increase in Apo E was observed in the VLDL + LDL fraction.

### Discussion

Alterations of cholesterol metabolism, including an increase of cholesterol synthesis and an accumulation of cholesterol esters in proliferating tissues associated with a decrease of HDL cholesterol in serum, were previously found in different experimental models of cell proliferation (Dessí *et al.*, 1984; 1986; 1989).

The present study confirms and extends these observations using as a model the rat ascites hepatoma AH-130, a rapidly growing lethal tumour.

In this model the increase of cholesterol synthesis was associated with a progressive accumulation of cholesterol in growing AH-130 cells. Initially, the accumulation was mostly due to an increased content in cholesterol esters, and later an increase in free cholesterol was also observed. These findings are consistent with several reports in the literature showing that, in a variety of tumours, cell membranes are enriched in free cholesterol (Feo *et al.*, 1973) and that cholesterol esters accumulate in tumour cells (Clayman *et al.*, 1986; Rao *et al.*, 1983) or in the proliferating tissue (Dessí *et al.*, 1984; 1986; Fex & Wallinder, 1973).

The biological significance of such phenomena remains to be established, nor is it clear whether the cholesterol accumulated in tumour tissues derives from new synthesis and/or increased uptake. However, it is likely that during processes of rapid cell proliferation, cholesterol esters are stored inside cells probably to meet the increased demand of cholesterol for new membrane biogenesis.

Alterations of intracellular cholesterol metabolism were accompanied by changes in total serum lipids and in lipoprotein profiles. All plasma lipid classes were elevated. This was due to an increase of lipid moietes in VLDL + LDL while phospholipid and cholesterol in HDL fractions were actually decreased.

Concomitantly, changes in host apoproteins were also observed: Apo E increased in VLDL + LDL fractions, while a decline in Apo AI and Apo AIV was observed in HDL lipoproteins. The latter observation suggests that changes in lipoprotein pattern in tumour-bearing rats may not be entirely explained by alterations in the amount of lipids bound to each lipoprotein class, but could also be related to changes in the absolute number of circulating lipoprotein particles.

The interpretation of our overall results is complicated because the distribution of lipids in the different classes of lipoproteins reflects the balance of lipids derived from endogenous biosynthesis, catabolism, diet, mobilisation of stored fat and the tumour itself.

In our model, tumour-bearing rats, developed a pronounced hypercholesterolemia and hypertriglyceridemia during the total period of tumour growth. At least a few possibilities can be considered to explain these findings: an increase mobilisation of lipids from fat depots as evidenced by the observed cachexia, a decreased catabolism of VLDL mediated by TNF (Ettinger *et al.*, 1990), being this vector elevated in serum during tumour growth and finally, since an increase in Apo E was also found, a decreased uptake by the liver of VLDL and LDL via Apo E receptors must be also considered. These three possibilities are not mutually exclusive.

It appears unlikely that changes in diet and endogenous biosynthesis may be involved under our experimental conditions. Hepatic cholesterol synthesis and food intake, the two main sources of plasma lipid under normal conditions, were in fact both decreased in tumour-bearing rats.

**Figure 1** Elution pattern of HDL apolipoproteins from normal and AH-130 tumour-bearing rats. Column: G3000SW (600 × 7.5 nm I.D.). Eluent: 0.1M sodium phosphate buffer (pH 7.0) containing 0.1% SDS. Flow rate: 0.30 ml min$^{-1}$. Load volume: 175 μl. Peaks: 1 = Apo AIV, 2 = Apo AI, 3 = Apo C. (**a**) normal rats; (**b**) 4 days after transplantation; (**c**) 7 days; (**d**) 10 days.

**Figure 2** Elution pattern of VLDL + LDL apolipoproteins from normal and AH-130 tumour-bearing rats. Column: G3000SW (600 × 7.5 nm I.D.). Eluent: 0.1M sodium phosphate buffer (pH 7.0) containing 0.1% SDS. Flow rate: 0.30 ml min$^{-1}$. Load volume: 175 μl. Peaks: 1 = Apo E, 2 = Apo C. (**a**) normal rats; (**b**) 4 days after tumour transplantation; (**c**) 7 days; (**d**) 10 days.

In this model, HDL steadily decreased over the course of tumour growth (4 and 7 days), then increased to near normal levels at 10 days, a time coinciding with the stationary phase of tumour growth. Thus it seems that the decrease in HDL levels may be a specific response to cell proliferation rather than directly related to the presence of tumour. A decrease of HDL was previously observed in our laboratories in different experimental models of cell proliferation, such as liver regeneration after partial hepatectomy (Dessí *et al*., 1986), bone marrow hyperplasia induced by phenylhydrazine (Dessí *et al*., 1990) and more recently in patients with different types of haematologic neoplasma (Dessí *et al*., 1991) and in G6PD deficient children with bone marrow hyperplasia after haemolysis induced by ingestion of fava bean (favism) (Dessí *et al*., 1992). In these models, however, the decrease of HDL was not associated with hyperlipidemia.

Taken together these findings suggest that the reported changes in total plasma lipid concentrations do not reflect a general pattern associated with growth, being variable and dependent on the type of hyperplastic or neoplastic growth. In contrast, the decrease in HDL fraction, virtually present in all models of cell proliferation, seems to represent a generalised phenomenon related to rapid cell proliferative processes.

A major function attributed to HDL is the ability to remove excess cholesterol from extrahepatic cells (Eisenberg, 1984). Since during proliferative processes the utilisation and storage of cholesterol are increased in proliferating tissues, it is possible to hypothesise that the observed decrease in HDL may be caused, at least partially, by a reduced release of free cholesterol from proliferating cells to HDL. Many studies *in vitro* support this conclusion: the exposure to HDL results in a net efflux of free cholesterol from various cultured cells (Daerr *et al*., 1980; Daniels *et al*., 1980), this efflux being partially blocked in rapidly proliferating cells and in transformed cell lines (Gebhard *et al*., 1987; Pittman *et al*., 1987).

Furthermore, Oram *et al*. (1987) have demonstrated that Apo AI-HDL binds to cell surface receptors and promotes selective removal of excess cholesterol from intracellular pool. The activity of these receptors is regulated by both the availability of exogenous cholesterol and the growth state of the cells.

Treatment of quiescent cells with serum growth factors suppresses both HDL receptor activity and HDL-mediated cholesterol efflux (Bierman *et al*., 1989). An opposite effect was obtained by the treatment of cultured fibroblasts with inhibitors of cell proliferation (Oppenheimer *et al*., 1988).

In line with these data, we have recently demonstrated that the inhibition *in vivo* of cholesterol esters accumulation by a specific inhibitor of ACAT, strongly prevents the decrease of HDL normally found during proliferative processes (Anchisi *et al*., (1990), giving support to the hypothesis that HDL alterations in serum are dependent on the altered cholesterol metabolism in proliferating tissues.

This work was supported by grants from CNR, Ministero dell 'Università e della Ricerca Scientifica e Tecnologica (40% e 60%), AIRC and Regione Autonoma della Sardegna.

### References

ANCHISI, C., BATETTA, B., DESSÍ, S., FADDA, M.F., MACCIONI, A. & PANI, P. (1990). Analisi HPLC di lipoproteine seriche di ratti trattati con piombo nitrato e clorpromazina. *Acta Tech. Legis Med*., **1**, 129-136.

BIERMAN, E.L., OPPENHEIMER, M. & ORAM, J.F. (1989). The regulation of HDL receptor activity. In Crepaldi, G., Gotto, A.M., Manzato, E. & Baggio, G. (eds) *Atherosclerosis VIII*, Excepta Medica: Amsterdam, New York, Oxford, pp. 297-300.

BOER, G.J. (1975). A simplified microassay of DNA and RNA using ethidium bromide. *Anal. Biochem*., **193**, 225-231.

BOWMAN, R.E. & WOLF, R.C. (1962). A rapid and specific ultramicromethod for total serum cholesterol. *Clin. Chem*., **8**, 302-309.

CLAYMAN, R.V., BILHARTZ, L.E., BUJA, M., SPADY, D.K. & DIETSCHY, J.M. (1986). Renal cell carcinoma in the Wistar-Lewis rat: a model for studying the mechanisms of cholesterol acquisition by the tumor *in vivo*. *Cancer Res*., **46**, 2958-2963.

COLEMAN, P.S. & LAVIETES, B.B. (1981). Membrane cholesterol tumorigenesis and the biochemical phenotype of neoplasia. *CRC Crit. Rev. Biochem*., **11**, 341-393.

DAERR, W.H., GIANTURCO, S.H., PATSCH, J.R., SMITH, L.C. & GOTTO, A.M.Jr (1980). Stimulation and suppression of 3-hydroxy-3-methyl-glutaryl coenzyme A reductase in normal human fibroblasts by high density lipoprotein subclasses. *Biochim. Biophys. Acta*, **619**, 287-301.

DANIELS, R.J., GUERTLER, L.S., PARCHER, T.S. & STEINBERG, D. (1980). Studies on the rate of efflux of cholesterol from cultured human skin fibroblasts. *J. Biol. Chem*., **256**, 4978-4983.

DESSÍ, S., BATETTA, B., LACONI, E., ENNAS, C. & PANI, P. (1984). Hepatic cholesterol in lead nitrate induced during liver hyperplasia. *Chem. Biol. Interact*., **48**, 271-279.

DESSÍ, S., CHIODINO, C., BATETTA, B., FADDA, A.M., ANCHISI, C. & PANI, P. (1986). Hepatic glucose-6-phosphate dehydrogenase, cholesterogenesis and serum lipoproteins in liver regeneration after partial hepatectomy. *Exp. Mol. Pathol*., **44**, 169-176.

DESSÍ, S., BATETTA, B., CARRUCCIU, A., PULISCI, D., LACONI, S., FADDA, A.M., ANCHISI, C. & PANI, P. (1989). Variations of serum lipoproteins during cell proliferation induced by lead nitrate. *Exp. Mol. Pathol*., **51**, 97-102.

DESSÍ, S., BATETTA, B., SPANO, O., PULISCI, D., ANCHISI, C., PANI, P. & BROCCIA, G. (1990). Serum lipoproteins during bone marrow hyperplasia after phenylhydrazine administration in rats. *Int. J. Exp. Pathol*., **74**, 671-675.

DESSÍ, S., BATETTA, B., PULISCI, D., ACCOGLI, P., PANI, P. & BROCCIA, G. (1991). Total and HDL cholesterol in human hematologic neoplasms. *Int. J. Hematol*., **54**, 483-486.

DESSÍ, S., BATETTA, B., SPANO, O., PULISCI, D., MULAS, M.F., MUNTONI, Sa., ARMENI, M., SANNA, C., ANTONUCCI, R. & PANI, P. (1992). Serum lipoprotein pattern as modified in G6PD-deficient children during haemolytic anaemia induced by fava bean ingestion. *Int. J. Exp. Pathol*., **73**, 157-160.

EISENBERG, S. (1984). High density lipoprotein metabolism. *J. Lipid. Res*., **25**, 1017-1058.

ERICKSON, S.K., COOPER, A.D., BARNARD, G.F., HAVEL, C.M., WATSON, J.A., FEINGOLD, K.R., MOSER, A.H., HUGHES-FULGORD, M. & SIPERSTEIN, M.D. (1988). Regulation of cholesterol metabolism in a slow-growing hepatoma *in vivo*. *Biochim. Biophys. Acta*, **960**, 131-138.

ETTINGER, W.H., MILLER, L.D., ALBERTS, J.J., SMITH, T.K. & PARKS, J.S. (1990). Lipopolysaccharide and tumor necrosis factor cause a fall in plasma concentration of lecithin: cholesterol acyltransferase in cynomolgus monkeys. *J. Lipid Res*., **31**, 1099-1107.

FEO, F., CANUTO, R.A., BERTONE, G., GARCEA, R. & PANI, P. (1973). Cholesterol and phospholipid composition of mitochondria and microsomes isolated from Morris hepatoma 5123 and rat liver. *FEBS Lett*., **23**, 229-232.

FEX, G. & WALLINDER, L. (1973). Liver and plasma cholesteryl ester metabolism after partial hepatectomy in the rat. *Biochim. Biophys. Acta*, **316**, 91-97.

FLICK, D.A. & GIFFORD, G.E. (1984). Comparison of *in vitro* cell cytotoxic assays for tumor necrosis factor. *J. Immunol. Methods*, **68**, 167-175.

GEBHARD, R.L., CLAYMAN, R.V., PRIGGE, W.F., FIGENSHAU, R., STALEY, N.A., REESEY, C. & BEAR, A. (1987). Abnormal cholesterol metabolism in renal clear cell carcinoma. *J. Lipid Res*., **28**, 1177-1184.

HABENICHT, A.J.R., GLOMSET, J.A. & ROSS, R. (1980). Relation of cholesterol and mevalonic acid to the cell cycle in smooth muscle and Swiss 3T3 cells stimulated to divide by platelet derived growth factor. *J. Biol. Chem*., **255**, 5134-5140.

HAVEL, R.J., EDER, H.A. & BRAGDEN, J.H. (1955). The distribution and chemical composition of ultracentrifugally separated lipoproteins in human serum. *J. Clin. Invest*., **34**, 1345-1353.

LOWRY, O.H., ROSEBROUGH, N.J., FARR, A.L. & RANDALL, R.J. (1951). Protein measurement with the Folin phenol reagent. *J. Biol. Chem*., **193**, 265-275.

OKAZAKY, M., KINOSHITA, M., NAITO, C. & HARA, I. (1984). High performance liquid chromatography of apolipoproteins in serum high-density lipoproteins. *J. Chromatogr*., **336**, 151-159.

OPPENHEIMER, M.J., ORAM, J.F. & BIERMAN, E.L. (1988). Up-regulation of high density lipoprotein receptor activity by interferon associated with inhibition of cell proliferation. *J. Biol. Chem.*, **263**, 19318–19323.

ORAM, J.F., JOHNSON, C. & BROWN, T.A. (1987). Interaction of high density lipoprotein with its receptor on cultured fibroblasts and macrophages. *J. Biol. Chem.*, **262**, 2405–2410.

PITTMAN, R.C., KNECHT, T.P., RESENBAUM, M.S. & TAYLOR, C.A.Jr. (1987). A nonendocytotic mechanism for the selective uptake of high density lipoprotein-associated cholesterol esters. *J. Biol. Chem.*, **262**, 2443–2450.

RAO, K.N., KOTTAPALLY, S. & SHINOZUKA, H. (1983). Lipid composition and 3-hydroxy-3-methylglutaryl-CoA reductase activity of acinar cell carcinoma of rat pancreas. *Biochim. Biophys. Acta*, **459**, 74–80.

SIPERSTEIN, M.D. (1984). Role of cholesterogenesis and isoprenoid synthesis in DNA replication and cell growth. *J. Lipid Res.*, **25**, 1462–1468.

TESSITORE, L., BONELLI, G. & BACCINO, F.M. (1987). Early development of protein metabolic perturbation in the liver and skeletal muscle of tumor-bearing rats. *Biochem. J.*, **241**, 153–159.

VAN BLITTERSWIJK, W.J., DAMEN, J., HILKMANN, H. & DE WIDT, J. (1985). Alterations in biosynthesis and homeostasis of cholesterol and in lipoprotein patterns in mice bearing a transplanted lymphoid tumor. *Biochim. Biophys. Acta*, **816**, 45–56.